# Safety and efficacy of active surveillance in patients with localized prostate cancer

**Published:** 2008

**Authors:** Ravimohan Mavuduru, Mayank Mohan Agarwal, Arup K. Mandal

**Affiliations:** Department of Urology, Level II, B Block, Advanced Urology Center, Nehru Hospital, Postgraduate Institute of Medical Education and Research, Chandigarh - 160 012, India. E-mail: drmayank_22@yahoo.co.in; drmayank.22@gmail.com

## SUMMARY

The authors analyzed baseline characteristics and outcome parameters of active surveillance in 278 men with prostate cancer screen-detected in the Rotterdam section of the European Randomized Study of Screening for Prostate Cancer (ERSPC) during 1993 to 2006. Recruitment and surveillance were not guided by protocol but depended on individual decisions of patients and their physicians. At diagnosis, the median age was 69.8 years (25-75 p; 66.1-72.8); median PSA 3.6 ng/ml (25-75 p; 3.1-4.8) and the clinical stage was T1c in 220 (79.1%) and T2 in 58 (20.9%). During the follow-up of median 3.4 years, 103 men (44.2%) had a PSA doubling time that was negative (i.e. half-life) or longer than 10 years. They found that men detected at rescreening were significantly more likely to be on active surveillance and they had more beneficial characteristics. Deferred treatment was elected in 82 cases (29.0%). Overall survival was 89% and disease-specific survival 100% after eight years.

## COMMENTS

There is a certain view of prostate cancer as a condition with which some patients live symbiotically, a condition that might never cause symptoms nor affect lifespan. This view was formed based on inferential evidence from the Veterans' 1967 studies,[1] which suggested that the early treatment of advanced prostate cancer was not thought to have a survival advantage. First described by Richard Choo (2001) from Toronto in a report of ‘watchful observation with selective delayed intervention for clinical, histologic or PSA progression’, active surveillance is an attractive approach in the management of early prostate cancer, which may spare men the side-effects of treatment, without compromising survival. Active surveillance also provides an ideal setting for research to identify new markers, which, in the future, could improve our ability to determine which men need treatment and which do not.

The index report shows a beneficial, although preliminary, outcome of screen-detected men managed on active surveillance. Although this study is a prospective randomized study it has some inherent flaws. The foremost was that the recruitment and the surveillance were based on individual decisions and not on any particular protocol. Only 15% of the screen-detected population opted for active surveillance which is a relatively small proportion and may not represent the entire populace. The article does not comment on the follow-up of those patients who did not opt for active surveillance. The report also shows that an important proportion of men have prolonged PSA doubling times, although the value of this parameter has not been established in untreated men. It would have been interesting to see the long-term follow-up, especially quality of life, of those patients who did not opt for active surveillance. Providing the data would have enabled us to further validate the utility of the role of active surveillance. Wong *et al.*,[2] performed a retrospective observational study of survival of 45000 men who were diagnosed with prostate cancer between 1991 and 1999, selected from the American Surveillance, Epidemiology and End Results (SEER) database. Patients were divided into groups who either had or did not have active treatment. They reported a significant difference in overall survival in favor of the treatment group, of which 23.8% of patients had died *vs*. 37% of the observation group. This translated as an overall improvement in survival in the treatment group of 30%. The benefit was greater for younger as compared with older men. Although patients who chose observation as their initial treatment felt less confident about their overall cancer control and felt less informed about their treatment choice, they expressed less regret about their decision than those who had surgery or radiotherapy.[3] Thus active surveillance does not affect the quality of life of the patients. It is hoped that active surveillance will avoid ‘unnecessary’ treatment and its associated side-effects, without detriment to long-term survival. However, it needs to be further verified by the results of the ongoing trials (The American Veterans' Affairs Prostate Cancer Intervention Versus Observation Trial (PIVOT), The UK Protect Study) comparing the survival of patients on active surveillance with those who received active treatment.
